# *N*,*N*′-Diphenyl-9,10-dioxo-9,10-di­hydro­anthracene-2,7-disulfonamide

**DOI:** 10.1107/S1600536813017303

**Published:** 2013-06-29

**Authors:** Wei-Guan Yuan, Hong-Ling Zhang

**Affiliations:** a Chemical Synthesis and Pollution Control Key Laboratory of Sichuan Province, School of Chemistry and Chemical Engineering, China West Normal University, Nanchong 637002, People’s Republic of China

## Abstract

The title mol­ecule, C_26_H_18_N_2_O_6_S_2_, has an overall Z-shaped conformation, in which the benzene rings are inclined to the anthra­quinone mean plane by 60.60 (9) and 50.66 (13)°. In the crystal, N—H⋯O and C—H⋯O hydrogen bonds link the mol­ecules into layers parallel to the *bc* plane.

## Related literature

For applications of sulfonamide derivitives, see: Valeur & Leray (2000 ▶); Chen *et al.* (2000 ▶); Kuljit & Subodh (2011 ▶). For applications of anthra­quinone derivitives, see: Lu *et al.* (2006 ▶); Liu *et al.* (2011 ▶). For details of the synthesis, see: Kuljit & Subodh (2011 ▶); Zeng & King (2002 ▶). For a related structure, see: Li *et al.* (2009 ▶). For standard bond lengths, see: Allen *et al.* (1987 ▶).
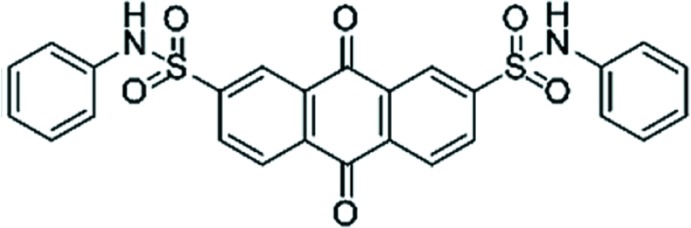


## Experimental

### 

#### Crystal data

C_26_H_18_N_2_O_6_S_2_
*M* *_r_* = 518.54
Monoclinic, 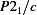

*a* = 10.247 (4) Å
*b* = 6.395 (2) Å
*c* = 36.265 (12) Å
β = 104.511 (12)°
*V* = 2300.6 (14) Å^3^
*Z* = 4
Mo *K*α radiation
μ = 0.28 mm^−1^
*T* = 293 K
0.26 × 0.17 × 0.14 mm

#### Data collection

Bruker APEXII CCD diffractometer
Absorption correction: multi-scan (*SADABS*; Bruker, 2008 ▶) *T*_min_ = 0.931, *T*_max_ = 0.962
12233 measured reflections
4512 independent reflections
1965 reflections with *I* > 2σ(*I*)
*R*_int_ = 0.088

#### Refinement

*R*[*F* ^2^ > 2σ(*F* ^2^)] = 0.059
*wR*(*F* ^2^) = 0.146
*S* = 1.00
4512 reflections
325 parameters
H-atom parameters constrained
Δρ_max_ = 0.33 e Å^−3^
Δρ_min_ = −0.32 e Å^−3^

### 

Data collection: *APEX2* (Bruker, 2008 ▶); cell refinement: *SAINT* (Bruker, 2008 ▶); data reduction: *SAINT*; program(s) used to solve structure: *SHELXS97* (Sheldrick, 2008 ▶); program(s) used to refine structure: *SHELXL97* (Sheldrick, 2008 ▶); molecular graphics: *SHELXTL* (Sheldrick, 2008 ▶); software used to prepare material for publication: *SHELXTL*.

## Supplementary Material

Crystal structure: contains datablock(s) global, I. DOI: 10.1107/S1600536813017303/cv5417sup1.cif

Structure factors: contains datablock(s) I. DOI: 10.1107/S1600536813017303/cv5417Isup2.hkl

Supplementary material file. DOI: 10.1107/S1600536813017303/cv5417Isup3.cml

Additional supplementary materials:  crystallographic information; 3D view; checkCIF report

## Figures and Tables

**Table 1:** Hydrogen-bond geometry (Å, °)

*D*—H⋯*A*	*D*—H	H⋯*A*	*D*⋯*A*	*D*—H⋯*A*
N2—H2*B*⋯O4^i^	0.86	2.55	3.110 (4)	123
N1—H1*B*⋯O2^ii^	0.86	2.32	2.952 (5)	131
C19—H19*A*⋯O6^iii^	0.93	2.35	3.164 (5)	146

Symmetry codes: (i) 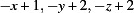; (ii) 
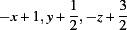
; (iii) 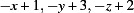.

**Fig. 1.**
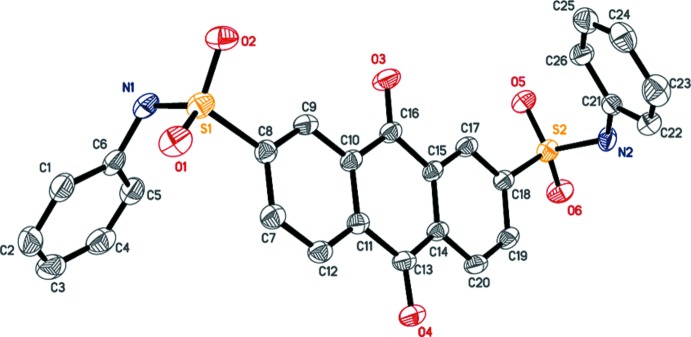
A view of the molecular structure of (I) with atom numbering. The displacement ellipsoids are drawn at the 30% probability level. H atoms omitted for clarity.

## supplementary crystallographic information

### Comment

In recent years, sulfonamide and its derivatives attract more attention due to their applications in molecular recognition (Kuljit *et al.*, 2011). They can be used as fluorescent sensors to detect heavy metal ions (Chen *et al.*, 2000) in view of good sensitivity, high selectivity, fast response and convenient observation (Valeur & Leray, 2000). Normally, a fluorescent sensor consists of a receptor for recognition and a fluorophore for signaling the recognition event. Anthraquinone and many of its derivatives are analogous to naphthalene dyes, therefore, they have been extensively explored as fluorescence probes in various chemical and biological systems (Lu *et al.*, 2006; Liu *et al.*, 2011). Herein, we report the synthesis and crystal structure of the title compound, (I), in which the anthraquinone fragment acts as a fluorophore.

In (I) (Fig. 1), the bond lengths and angles are normal (Allen *et al.*, 1987) and correspond well to those observed in the related compound (Li *et al.*, 2009). Two benzene rings are inclined to the anthraquinone mean plane at 60.60 (9)° and 50.66 (13)°, respectively. In the crystal, intermolecular N—H···O and C—H···O hydrogen bonds (Table 1) link the molecules into layers parallel to *bc* plane.

### Experimental

A mixture of aniline (372 mg, 4 mmol) and triethylamine(8 mmol) in dry dichloroethane(20 ml) was stirred at RT, then, the solution of N,*N*^'^– bisphenyl- 9,10- dioxo-9,10-dihydro-2,7- anthracenedisulfonyl chloride (977 mg, 2.2 mmol) in dry dichloroethane (30 ml) was added during 5 min, the mixture was stirred at RT for 8 h under nitrogen in air. Then the solvent was removed completely under vacuum and the solid washed with water yielding the title compound (Kuljit *et al.*,2011; Zeng *et al.*,2002). Yellow needlelike single crystals suitable for X-ray diffraction were obtained by volatilizing dichloromethane slowly.

### Refinement

H atoms were placed in calculated positions [N—H = 0.86 Å, C—H = 0.93 Å], and refined in riding mode, with *U*_iso_(H) = 1.2*U*_eq_(C, N).

### Crystal data

**Table:**

C_26_H_18_N_2_O_6_S_2_	*F*(000) = 1072
*M**_r_* = 518.54	*D*_x_ = 1.497 Mg m^−^^3^
Monoclinic, *P*2_1_/*c*	Mo *K*α radiation, λ = 0.71073 Å
Hall symbol: -P 2ybc	Cell parameters from 620 reflections
*a* = 10.247 (4) Å	θ = 2.7–17.7°
*b* = 6.395 (2) Å	µ = 0.28 mm^−^^1^
*c* = 36.265 (12) Å	*T* = 293 K
β = 104.511 (12)°	Needle, yellow
*V* = 2300.6 (14) Å^3^	0.26 × 0.17 × 0.14 mm
*Z* = 4	

### Data collection

**Table:**

Bruker APEXII CCD diffractometer	4512 independent reflections
Radiation source: fine-focus sealed tube	1965 reflections with *I* > 2σ(*I*)
Graphite monochromator	*R*_int_ = 0.088
φ and ω scans	θ_max_ = 26.0°, θ_min_ = 1.2°
Absorption correction: multi-scan (*SADABS*; Bruker, 2008)	*h* = −8→12
*T*_min_ = 0.931, *T*_max_ = 0.962	*k* = −7→7
12233 measured reflections	*l* = −44→44

### Refinement

**Table:**

Refinement on *F*^2^	Primary atom site location: structure-invariant direct methods
Least-squares matrix: full	Secondary atom site location: difference Fourier map
*R*[*F*^2^ > 2σ(*F*^2^)] = 0.059	Hydrogen site location: inferred from neighbouring sites
*wR*(*F*^2^) = 0.146	H-atom parameters constrained
*S* = 1.00	*w* = 1/[σ^2^(*F*_o_^2^) + (0.040*P*)^2^] where *P* = (*F*_o_^2^ + 2*F*_c_^2^)/3
4512 reflections	(Δ/σ)_max_ < 0.001
325 parameters	Δρ_max_ = 0.33 e Å^−^^3^
0 restraints	Δρ_min_ = −0.32 e Å^−^^3^

### Special details

**Table:**

Geometry. All e.s.d.'s (except the e.s.d. in the dihedral angle between two l.s. planes) are estimated using the full covariance matrix. The cell e.s.d.'s are taken into account individually in the estimation of e.s.d.'s in distances, angles and torsion angles; correlations between e.s.d.'s in cell parameters are only used when they are defined by crystal symmetry. An approximate (isotropic) treatment of cell e.s.d.'s is used for estimating e.s.d.'s involving l.s. planes.
Refinement. Refinement of *F*^2^ against ALL reflections. The weighted *R*-factor *wR* and goodness of fit *S* are based on *F*^2^, conventional *R*-factors *R* are based on *F*, with *F* set to zero for negative *F*^2^. The threshold expression of *F*^2^ > σ(*F*^2^) is used only for calculating *R*-factors(gt) *etc*. and is not relevant to the choice of reflections for refinement. *R*-factors based on *F*^2^ are statistically about twice as large as those based on *F*, and *R*- factors based on ALL data will be even larger.

### Fractional atomic coordinates and isotropic or equivalent isotropic displacement parameters (Å^2^)

**Table:**

	*x*	*y*	*z*	*U*_iso_*/*U*_eq_	
S1	0.58554 (13)	0.18230 (18)	0.79332 (3)	0.0572 (4)	
S2	0.28393 (12)	1.46829 (17)	0.92991 (3)	0.0563 (4)	
O1	0.6506 (3)	−0.0105 (4)	0.80672 (8)	0.0677 (9)	
O2	0.4492 (3)	0.1852 (4)	0.77093 (8)	0.0677 (9)	
O3	0.3522 (3)	0.8677 (5)	0.83377 (8)	0.0714 (10)	
O4	0.7155 (3)	0.6645 (5)	0.96336 (7)	0.0645 (9)	
O5	0.2258 (3)	1.5295 (4)	0.89183 (7)	0.0634 (9)	
O6	0.3526 (3)	1.6176 (4)	0.95722 (8)	0.0720 (9)	
N1	0.6729 (4)	0.3019 (5)	0.76842 (9)	0.0580 (10)	
H1B	0.6322	0.3522	0.7466	0.070*	
N2	0.1695 (3)	1.3670 (5)	0.94892 (9)	0.0566 (10)	
H2B	0.1538	1.4248	0.9688	0.068*	
C1	0.8998 (6)	0.1581 (8)	0.77943 (12)	0.0693 (14)	
H1A	0.8636	0.0292	0.7703	0.083*	
C2	1.0385 (7)	0.1863 (11)	0.79121 (14)	0.0858 (17)	
H2A	1.0954	0.0752	0.7896	0.103*	
C3	1.0928 (6)	0.3734 (12)	0.80505 (14)	0.0867 (18)	
H3A	1.1858	0.3900	0.8129	0.104*	
C4	1.0079 (7)	0.5385 (10)	0.80733 (13)	0.0823 (16)	
H4A	1.0444	0.6671	0.8165	0.099*	
C5	0.8700 (6)	0.5145 (8)	0.79609 (12)	0.0665 (13)	
H5A	0.8138	0.6253	0.7984	0.080*	
C6	0.8160 (5)	0.3270 (8)	0.78157 (11)	0.0556 (12)	
C7	0.6861 (4)	0.2840 (7)	0.86814 (11)	0.0547 (12)	
H7A	0.7385	0.1643	0.8693	0.066*	
C8	0.5946 (4)	0.3395 (7)	0.83429 (11)	0.0472 (11)	
C9	0.5143 (4)	0.5151 (6)	0.83231 (11)	0.0481 (11)	
H9A	0.4528	0.5501	0.8096	0.058*	
C10	0.5262 (4)	0.6392 (6)	0.86460 (11)	0.0455 (11)	
C11	0.6200 (4)	0.5845 (6)	0.89853 (11)	0.0452 (11)	
C12	0.6987 (4)	0.4077 (7)	0.90007 (12)	0.0512 (11)	
H12A	0.7603	0.3719	0.9227	0.061*	
C13	0.6374 (4)	0.7168 (7)	0.93335 (12)	0.0489 (11)	
C14	0.5571 (4)	0.9119 (6)	0.93028 (11)	0.0455 (11)	
C15	0.4591 (4)	0.9599 (6)	0.89736 (11)	0.0438 (10)	
C16	0.4378 (4)	0.8243 (7)	0.86281 (11)	0.0502 (11)	
C17	0.3778 (4)	1.1325 (7)	0.89652 (11)	0.0503 (11)	
H17A	0.3115	1.1640	0.8746	0.060*	
C18	0.3949 (4)	1.2593 (6)	0.92850 (11)	0.0478 (11)	
C19	0.4951 (4)	1.2139 (7)	0.96133 (11)	0.0551 (12)	
H19A	0.5071	1.2998	0.9826	0.066*	
C20	0.5760 (4)	1.0420 (7)	0.96214 (11)	0.0538 (12)	
H20A	0.6434	1.0121	0.9839	0.065*	
C21	0.0947 (4)	1.1849 (7)	0.93340 (12)	0.0488 (11)	
C22	0.0754 (5)	1.0319 (8)	0.95845 (12)	0.0642 (13)	
H22A	0.1091	1.0515	0.9845	0.077*	
C23	0.0068 (5)	0.8506 (8)	0.94520 (17)	0.0824 (16)	
H23A	−0.0066	0.7488	0.9622	0.099*	
C24	−0.0416 (5)	0.8218 (8)	0.90673 (17)	0.0739 (15)	
H24A	−0.0871	0.6993	0.8975	0.089*	
C25	−0.0229 (5)	0.9733 (8)	0.88214 (14)	0.0710 (14)	
H25A	−0.0551	0.9522	0.8561	0.085*	
C26	0.0431 (4)	1.1582 (8)	0.89523 (12)	0.0626 (13)	
H26A	0.0521	1.2626	0.8782	0.075*	

### Atomic displacement parameters (Å^2^)

**Table:**

	*U*^11^	*U*^22^	*U*^33^	*U*^12^	*U*^13^	*U*^23^
S1	0.0700 (9)	0.0481 (7)	0.0503 (7)	−0.0020 (7)	0.0094 (6)	−0.0015 (6)
S2	0.0659 (8)	0.0510 (7)	0.0529 (8)	−0.0088 (7)	0.0166 (6)	−0.0050 (6)
O1	0.095 (2)	0.0398 (18)	0.0637 (19)	0.0054 (17)	0.0110 (18)	0.0079 (15)
O2	0.065 (2)	0.061 (2)	0.0660 (19)	−0.0071 (17)	−0.0047 (17)	−0.0067 (16)
O3	0.083 (2)	0.079 (2)	0.0415 (18)	0.0234 (19)	−0.0047 (17)	−0.0046 (16)
O4	0.066 (2)	0.078 (2)	0.0431 (18)	0.0071 (18)	0.0017 (16)	0.0034 (16)
O5	0.081 (2)	0.0580 (19)	0.0484 (18)	0.0018 (17)	0.0117 (17)	0.0043 (15)
O6	0.086 (2)	0.057 (2)	0.066 (2)	−0.0179 (18)	0.0065 (18)	−0.0195 (16)
N1	0.076 (3)	0.061 (2)	0.0359 (19)	0.001 (2)	0.013 (2)	0.0038 (18)
N2	0.063 (2)	0.062 (2)	0.051 (2)	−0.006 (2)	0.027 (2)	−0.0133 (18)
C1	0.087 (4)	0.074 (4)	0.048 (3)	0.012 (3)	0.021 (3)	−0.002 (3)
C2	0.088 (5)	0.118 (5)	0.059 (3)	0.035 (4)	0.032 (3)	0.017 (4)
C3	0.071 (4)	0.138 (6)	0.050 (3)	−0.007 (4)	0.014 (3)	0.018 (4)
C4	0.093 (5)	0.099 (5)	0.053 (3)	−0.012 (4)	0.015 (3)	0.006 (3)
C5	0.083 (4)	0.065 (4)	0.054 (3)	0.007 (3)	0.022 (3)	0.001 (3)
C6	0.079 (4)	0.054 (3)	0.035 (2)	0.007 (3)	0.018 (2)	0.001 (2)
C7	0.057 (3)	0.056 (3)	0.051 (3)	−0.001 (2)	0.012 (2)	0.002 (2)
C8	0.048 (3)	0.051 (3)	0.045 (3)	−0.006 (2)	0.015 (2)	−0.001 (2)
C9	0.047 (3)	0.052 (3)	0.041 (2)	−0.004 (2)	0.003 (2)	0.005 (2)
C10	0.051 (3)	0.049 (3)	0.036 (2)	−0.006 (2)	0.011 (2)	0.004 (2)
C11	0.045 (3)	0.049 (3)	0.041 (3)	−0.009 (2)	0.011 (2)	0.006 (2)
C12	0.048 (3)	0.060 (3)	0.044 (3)	−0.006 (2)	0.008 (2)	0.007 (2)
C13	0.045 (3)	0.057 (3)	0.041 (3)	−0.009 (2)	0.004 (2)	0.008 (2)
C14	0.046 (3)	0.051 (3)	0.037 (2)	−0.011 (2)	0.007 (2)	0.004 (2)
C15	0.052 (3)	0.044 (3)	0.036 (2)	−0.009 (2)	0.013 (2)	0.004 (2)
C16	0.055 (3)	0.056 (3)	0.038 (2)	−0.001 (2)	0.009 (2)	0.005 (2)
C17	0.054 (3)	0.056 (3)	0.040 (3)	−0.010 (2)	0.008 (2)	0.005 (2)
C18	0.050 (3)	0.050 (3)	0.044 (3)	−0.011 (2)	0.010 (2)	0.000 (2)
C19	0.066 (3)	0.058 (3)	0.041 (3)	−0.013 (3)	0.012 (2)	−0.009 (2)
C20	0.053 (3)	0.064 (3)	0.039 (3)	−0.007 (3)	0.001 (2)	0.000 (2)
C21	0.045 (3)	0.051 (3)	0.051 (3)	−0.001 (2)	0.013 (2)	−0.002 (2)
C22	0.064 (3)	0.077 (4)	0.051 (3)	−0.002 (3)	0.012 (3)	0.000 (3)
C23	0.085 (4)	0.072 (4)	0.095 (4)	−0.011 (3)	0.032 (4)	0.017 (3)
C24	0.065 (4)	0.060 (3)	0.100 (4)	−0.012 (3)	0.028 (3)	−0.005 (3)
C25	0.063 (3)	0.078 (4)	0.073 (3)	−0.010 (3)	0.019 (3)	−0.014 (3)
C26	0.064 (3)	0.070 (3)	0.052 (3)	−0.008 (3)	0.010 (3)	−0.002 (3)

### Geometric parameters (Å, º)

**Table:**

S1—O1	1.428 (3)	C9—C10	1.394 (5)
S1—O2	1.430 (3)	C9—H9A	0.9300
S1—N1	1.615 (4)	C10—C11	1.402 (5)
S1—C8	1.777 (4)	C10—C16	1.482 (6)
S2—O5	1.415 (3)	C11—C12	1.382 (5)
S2—O6	1.428 (3)	C11—C13	1.493 (5)
S2—N2	1.636 (3)	C12—H12A	0.9300
S2—C18	1.764 (4)	C13—C14	1.483 (5)
O3—C16	1.222 (4)	C14—C15	1.388 (5)
O4—C13	1.225 (4)	C14—C20	1.398 (5)
N1—C6	1.433 (5)	C15—C17	1.379 (5)
N1—H1B	0.8600	C15—C16	1.494 (5)
N2—C21	1.429 (5)	C17—C18	1.390 (5)
N2—H2B	0.8600	C17—H17A	0.9300
C1—C2	1.390 (7)	C18—C19	1.394 (5)
C1—C6	1.394 (6)	C19—C20	1.372 (5)
C1—H1A	0.9300	C19—H19A	0.9300
C2—C3	1.361 (7)	C20—H20A	0.9300
C2—H2A	0.9300	C21—C26	1.363 (5)
C3—C4	1.383 (7)	C21—C22	1.383 (6)
C3—H3A	0.9300	C22—C23	1.379 (6)
C4—C5	1.378 (6)	C22—H22A	0.9300
C4—H4A	0.9300	C23—C24	1.371 (6)
C5—C6	1.370 (6)	C23—H23A	0.9300
C5—H5A	0.9300	C24—C25	1.362 (6)
C7—C12	1.382 (5)	C24—H24A	0.9300
C7—C8	1.391 (5)	C25—C26	1.386 (6)
C7—H7A	0.9300	C25—H25A	0.9300
C8—C9	1.384 (5)	C26—H26A	0.9300
			
O1—S1—O2	120.75 (19)	C12—C11—C10	120.3 (4)
O1—S1—N1	108.8 (2)	C12—C11—C13	119.0 (4)
O2—S1—N1	106.02 (19)	C10—C11—C13	120.7 (4)
O1—S1—C8	106.36 (19)	C11—C12—C7	120.3 (4)
O2—S1—C8	107.9 (2)	C11—C12—H12A	119.9
N1—S1—C8	106.22 (19)	C7—C12—H12A	119.9
O5—S2—O6	120.37 (19)	O4—C13—C14	121.6 (4)
O5—S2—N2	110.50 (18)	O4—C13—C11	120.6 (4)
O6—S2—N2	104.67 (19)	C14—C13—C11	117.8 (4)
O5—S2—C18	107.33 (19)	C15—C14—C20	120.0 (4)
O6—S2—C18	108.5 (2)	C15—C14—C13	121.3 (4)
N2—S2—C18	104.34 (18)	C20—C14—C13	118.7 (4)
C6—N1—S1	122.1 (3)	C17—C15—C14	120.0 (4)
C6—N1—H1B	119.0	C17—C15—C16	119.0 (4)
S1—N1—H1B	119.0	C14—C15—C16	121.0 (4)
C21—N2—S2	121.8 (3)	O3—C16—C10	121.2 (4)
C21—N2—H2B	119.1	O3—C16—C15	120.9 (4)
S2—N2—H2B	119.1	C10—C16—C15	117.9 (4)
C2—C1—C6	118.6 (5)	C15—C17—C18	120.0 (4)
C2—C1—H1A	120.7	C15—C17—H17A	120.0
C6—C1—H1A	120.7	C18—C17—H17A	120.0
C3—C2—C1	121.3 (6)	C17—C18—C19	120.2 (4)
C3—C2—H2A	119.3	C17—C18—S2	121.2 (3)
C1—C2—H2A	119.3	C19—C18—S2	118.4 (3)
C2—C3—C4	119.2 (6)	C20—C19—C18	119.8 (4)
C2—C3—H3A	120.4	C20—C19—H19A	120.1
C4—C3—H3A	120.4	C18—C19—H19A	120.1
C5—C4—C3	120.7 (6)	C19—C20—C14	120.1 (4)
C5—C4—H4A	119.6	C19—C20—H20A	120.0
C3—C4—H4A	119.6	C14—C20—H20A	120.0
C6—C5—C4	119.8 (5)	C26—C21—C22	119.7 (4)
C6—C5—H5A	120.1	C26—C21—N2	122.4 (4)
C4—C5—H5A	120.1	C22—C21—N2	117.9 (4)
C5—C6—C1	120.3 (5)	C23—C22—C21	120.7 (4)
C5—C6—N1	120.5 (5)	C23—C22—H22A	119.7
C1—C6—N1	119.2 (5)	C21—C22—H22A	119.7
C12—C7—C8	119.6 (4)	C24—C23—C22	119.4 (5)
C12—C7—H7A	120.2	C24—C23—H23A	120.3
C8—C7—H7A	120.2	C22—C23—H23A	120.3
C9—C8—C7	120.9 (4)	C25—C24—C23	119.8 (5)
C9—C8—S1	120.7 (3)	C25—C24—H24A	120.1
C7—C8—S1	118.4 (3)	C23—C24—H24A	120.1
C8—C9—C10	119.5 (4)	C24—C25—C26	121.3 (5)
C8—C9—H9A	120.3	C24—C25—H25A	119.4
C10—C9—H9A	120.3	C26—C25—H25A	119.4
C9—C10—C11	119.5 (4)	C21—C26—C25	119.1 (5)
C9—C10—C16	119.6 (4)	C21—C26—H26A	120.4
C11—C10—C16	121.0 (4)	C25—C26—H26A	120.4
			
O1—S1—N1—C6	−47.7 (4)	C11—C13—C14—C15	6.4 (6)
O2—S1—N1—C6	−179.0 (3)	O4—C13—C14—C20	2.6 (6)
C8—S1—N1—C6	66.5 (4)	C11—C13—C14—C20	−177.5 (4)
O5—S2—N2—C21	60.2 (3)	C20—C14—C15—C17	−1.9 (6)
O6—S2—N2—C21	−168.9 (3)	C13—C14—C15—C17	174.1 (4)
C18—S2—N2—C21	−54.9 (3)	C20—C14—C15—C16	179.5 (4)
C6—C1—C2—C3	0.8 (7)	C13—C14—C15—C16	−4.5 (6)
C1—C2—C3—C4	−0.1 (8)	C9—C10—C16—O3	1.8 (6)
C2—C3—C4—C5	0.6 (8)	C11—C10—C16—O3	−177.0 (4)
C3—C4—C5—C6	−1.7 (7)	C9—C10—C16—C15	−176.6 (4)
C4—C5—C6—C1	2.4 (7)	C11—C10—C16—C15	4.7 (6)
C4—C5—C6—N1	−177.6 (4)	C17—C15—C16—O3	2.0 (6)
C2—C1—C6—C5	−1.9 (7)	C14—C15—C16—O3	−179.4 (4)
C2—C1—C6—N1	178.1 (4)	C17—C15—C16—C10	−179.7 (4)
S1—N1—C6—C5	−102.5 (4)	C14—C15—C16—C10	−1.1 (6)
S1—N1—C6—C1	77.5 (5)	C14—C15—C17—C18	0.6 (6)
C12—C7—C8—C9	−1.1 (6)	C16—C15—C17—C18	179.3 (4)
C12—C7—C8—S1	177.7 (3)	C15—C17—C18—C19	0.7 (6)
O1—S1—C8—C9	−164.6 (3)	C15—C17—C18—S2	−174.6 (3)
O2—S1—C8—C9	−33.8 (4)	O5—S2—C18—C17	−24.7 (4)
N1—S1—C8—C9	79.5 (4)	O6—S2—C18—C17	−156.2 (3)
O1—S1—C8—C7	16.5 (4)	N2—S2—C18—C17	92.6 (4)
O2—S1—C8—C7	147.4 (3)	O5—S2—C18—C19	160.0 (3)
N1—S1—C8—C7	−99.3 (3)	O6—S2—C18—C19	28.4 (4)
C7—C8—C9—C10	0.7 (6)	N2—S2—C18—C19	−82.7 (3)
S1—C8—C9—C10	−178.1 (3)	C17—C18—C19—C20	−0.7 (6)
C8—C9—C10—C11	0.3 (6)	S2—C18—C19—C20	174.7 (3)
C8—C9—C10—C16	−178.4 (4)	C18—C19—C20—C14	−0.6 (6)
C9—C10—C11—C12	−0.8 (6)	C15—C14—C20—C19	1.9 (6)
C16—C10—C11—C12	177.9 (4)	C13—C14—C20—C19	−174.2 (4)
C9—C10—C11—C13	178.6 (4)	S2—N2—C21—C26	−43.2 (5)
C16—C10—C11—C13	−2.7 (6)	S2—N2—C21—C22	136.7 (3)
C10—C11—C12—C7	0.3 (6)	C26—C21—C22—C23	1.3 (7)
C13—C11—C12—C7	−179.1 (4)	N2—C21—C22—C23	−178.6 (4)
C8—C7—C12—C11	0.6 (6)	C21—C22—C23—C24	0.6 (7)
C12—C11—C13—O4	−3.5 (6)	C22—C23—C24—C25	−0.8 (8)
C10—C11—C13—O4	177.2 (4)	C23—C24—C25—C26	−0.7 (8)
C12—C11—C13—C14	176.6 (4)	C22—C21—C26—C25	−2.8 (7)
C10—C11—C13—C14	−2.8 (6)	N2—C21—C26—C25	177.0 (4)
O4—C13—C14—C15	−173.5 (4)	C24—C25—C26—C21	2.6 (7)

### Hydrogen-bond geometry (Å, º)

**Table:**

*D*—H···*A*	*D*—H	H···*A*	*D*···*A*	*D*—H···*A*
N2—H2*B*···O4^i^	0.86	2.55	3.110 (4)	123
N1—H1*B*···O2^ii^	0.86	2.32	2.952 (5)	131
C19—H19*A*···O6^iii^	0.93	2.35	3.164 (5)	146

Symmetry codes: (i) −*x*+1, −*y*+2, −*z*+2; (ii) −*x*+1, *y*+1/2, −*z*+3/2; (iii) −*x*+1, −*y*+3, −*z*+2.
